# Knowledge of the Unknown Child: A Systematic Review of the Elements of the Best Interests of the Child Assessment for Recently Arrived Refugee Children

**DOI:** 10.1007/s10567-016-0209-y

**Published:** 2016-07-07

**Authors:** E. C. C. van Os, M. E. Kalverboer, A. E. Zijlstra, W. J. Post, E. J. Knorth

**Affiliations:** 1 Study Centre for Children, Migration and Law, Department of Special Needs Education and Youth Care, Faculty of Behavioural and Social Sciences, University of Groningen, Grote Rozenstraat 38, 9712 TJ Groningen, The Netherlands; 2 Department of Special Needs Education and Youth Care, University of Groningen, Groningen, The Netherlands

**Keywords:** Refugee children, Mental health, Best interests of the child assessment, Decision-making, Asylum

## Abstract

Decision-making regarding an asylum request of a minor requires decision-makers to determine the best interests of the child when the minor is relatively unknown. This article presents a systematic review of the existing knowledge of the situation of recently arrived refugee children in the host country. This research is based on the General Comment No. 14 of UN Committee on the Rights of the Child. It shows the importance of knowing the type and number of stressful life events a refugee child has experienced before arrival, as well as the duration and severity of these events. The most common mental health problems children face upon arrival in the host country are PTSD, depression and various anxiety disorders. The results identify the relevant elements of the best interests of the child assessment, including implications for procedural safeguards, which should promote a child rights-based decision in the asylum procedure.

## Introduction

Children on the move, fleeing from one country to another, leaving an unsafe but familiar environment and looking for safety in a new country, enter a decision-making procedure. Since countries have migration policies, children cannot simply cross a border to reach a place that is considered safer. The host country has to decide whether or not the child—travelling alone or with family members—will be accepted as a new citizen, temporary or permanently, i.e. as a refugee or as a child in need of other forms of protection. If the host country decides that the child is not entitled to a residence permit, the child will have to leave voluntarily or else will be deported. In taking that decision, the best interests of the child should be a primary consideration. This principle and substantive right is laid down in article 3 of the Convention on the Rights of the Child (CRC) (UN 1989).

### Determination of the Best Interests of the Child

The United Nations Committee on the Rights of the Child (2013) (hereafter the Committee) provides a tool for the assessment and determination of the best interests of the child in General Comment no. 14 (hereafter: GC 14). The Committee describes a non-exhaustive list of areas of concern that should be part of every best interests assessment:

- The child’s views; children should influence the determination of the best interests by expressing their views on the decision that affects them (GC 14, para. 53–54);
- The child’s identity, which includes characteristics such as cultural identity, religion, beliefs, sexual orientation and personality (GC 14, para. 55–57);
- Preservation of family environment and maintaining relations, which includes both the prevention of separation with the parents unless this is in the best interests of the child, and the preservation of the child’s ties beyond family, e.g. school and friends (GC 14, para. 58–70);
- Care, protection and safety of the child, necessary to ensure the child’s well-being, including emotional care and calculation of future risks and harm as a consequences of the decision (GC 14, para. 71–74);
- The state of vulnerability, such as being disabled, belonging to a minority group, being a refugee or victim of abuse, is to be assessed through the child’s history from birth (GC 14, para. 75–76);
- The child’s right to health (GC 14, para. 77–78); and.
- The child’s right to education (GC 14, para. 79).

Following these guidelines of the Committee, decision-making in a migration procedure obliges the decision-makers to gather a lot of information on an unknown—recently arrived—child and requires the decision-makers to be able to interpret this information in a way which corresponds with the best interests of the child principle. Therefore, the Committee advises to involve professionals trained in, inter alia, child psychology, child development and other relevant human and social development fields, who are experienced in working with children, and will consider the information received in an objective manner (GC 14, para. 94). Decision-making should be based on scientific knowledge (GC 14, para. 95). Inspired by this recommendation of the Committee, we will present a systematic review of the existing scientific knowledge in the field of social and behavioural sciences regarding recently arrived refugee children.

This paper focuses on both unaccompanied children and children who are accompanied by (one of) their parents or caregivers, who are *forced* to leave their home country in search of protection in another country. In most cases, these children ask for asylum and can therefore be defined in a legal sense as asylum-seeking children. Legally, these children are called refugees once their asylum claim has been accepted. Working from our pedagogical point of view, we prefer to call these children *refugees*: seeking protection either on the grounds of being a refugee in the sense of the 1951 Refugee Convention or because of other forms of perceived danger in the home country (UNHCR 1951).

Refugee children are considered vulnerable (Bean et al. 2007a, b; Huemer et al. 2013; Oppedal and Idsoe 2012; Thommessen et al. 2013; Vervliet et al. 2014a, b, c). Migration in itself may have a negative impact on the health, development and well-being of children (Abebe et al. 2014; Belhadj Kouider et al. 2014). Children who are *forced* to leave their home country due to war or other forms of violence are at an increased risk, as a result of the stressful events they may have experienced before and during the flight and uncertainty about their new home and future perspectives (Bronstein and Montgomery 2011; Fazel et al. 2012).

Much research has already been done with regard to the *mental health and development* of refugee children residing several years in the host country (Almqvist and Broberg 1999; Bean 2006; Bean et al. 2007b; Beiser et al. 2012; Dura-Vila et al. 2013; Geltman et al. 2005; Kalverboer et al. 2009; Lauritzen and Sivertsen 2012; Montgomery 2010; Oppedal and Idsoe 2012; Seglem et al. 2011; Vervliet et al. 2014a). These studies can show us some of the elements that play a role in the best interests assessment for *recently arrived* children as well. In two systematic reviews of the mental health of refugee children, the following risk factors—related to the pre- or during migration period—were identified: exposure to violence, personal injury, pre-existing vulnerability (cumulative), family experience of adverse events, unaccompanied entry and separation from parents or other relatives in the home country, the violent death of a family member and poor parental support or family cohesion (Bronstein and Montgomery 2011; Fazel et al. 2012). Knowledge of which risk factors apply to a child is necessary to estimate his or her level of vulnerability, one of the key elements of the best interests of the child assessment (GC 14, para. 75–76).

The *physicalhealth* of recently arrived refugee children is beyond the scope of our review. However, the condition of the child’s physical health should be part of the best interests of the child assessment (GC 14, para. 77). Moreover, the Committee explicitly mentions the need to consider the health of the child with regard to decisions such as granting a residence permit on humanitarian grounds (GC 14, para. 78). Excellent reviews are available on the physical health of refugee children upon arrival in the host country (Davidson et al. 2004; Raman et al. 2009; Sheikh et al. 2009).

The Committee recognizes both the individual characteristics of the child and the social-cultural context in which the child lives as the two pillars of the best interests of the child assessment. Examples of the relevant aspects of the social-cultural context are: the presence or absence of parents, the relationship between the child and the family members or other caregivers and the safety of the environment (GC 14, para. 48).

### Best Interests of the Child (BIC)-Model

The importance of a detailed analysis of the child’s family and social context as a base for decision-making has been recognized for many years in the study on the Best Interests of the Child-Model (Kalverboer et al. 2009; Kalverboer 2014; Kalverboer and Zijlstra 2006; Zijlstra 2012; a, b). The BIC-Model consists of fourteen pedagogical environmental conditions that promote and should safeguard the development of the child. The *right to development* is phrased in article six of the CRC and closely linked to the best interests concept. Moreover, States have the obligation to ensure this *right to development* in the assessment of the best interests of the child (GC 14, para. 42).

The first seven conditions in the BIC-Model that promote the child’s development concern the *family situation*: “Adequate physical care” (1), “Safe direct physical environment” (2), “Affective atmosphere” (3), “Supportive, flexible childrearing structure” (4), “Adequate example by parents” (5), “Interest” (6), and “Continuity in upbringing conditions, future perspective” (7). The other seven conditions refer to the *social environment* of the child: “Safe wider physical environment” (8), “Respect” (9), “Social network” (10), “Education” (11), “Contact with peers” (12), “Respect” (13) and “Stability in living circumstances” (14). See Table 1 for the definitions of these conditions and the relation between General Comment no. 14 and the conditions of the BIC-Model.

**Table 1:** The Best Interests of the Child-Model with references to the related articles in the Convention on the Rights of the Child (CRC) and to the paragraphs of the General Comment No. 14 (GC 14) of the UN Committee on the Rights of the Child on the best interests of the child assessment and determination

Family	Society
*Best Interests of the Child-Model*	
*Current situation*	
*1. Adequate physical care* Adequate physical care refers to the care for the child’s health and physical well-being by parents or care-providers. They offer the child a place to live, clothing to wear, enough food to eat and (some) personal belongings. There is a family income to provide for all this. In addition, the parents or care–providers are free of worries about providing for the child’s physical well-being CRC Art. 24, 26, 27 GC 14 para. 70, 71, 77, 78, 84	*8. Safe wider physical environment* The neighbourhood the child grows up in is safe, as well as the society the child lives in. Criminality, (civil) wars, natural disasters, infectious diseases etc. Do not threaten the development of the child CRC Art. 33, 34, 35, 36, 37 GC 14 para. 70, 71, 73, 74, 77, 78, 84
*2. Safe direct physical environment* A safe direct physical environment offers the child physical protection. This implies the absence of physical danger in the house or neighbourhood in which the child lives. There are no toxics or other threats in the house or neighbourhood. The child is not threatened by abuse of any kind CRC Art. 19, 24 GC 14 para 61, 70, 71, 73, 74, 77, 78, 84	*9. Respect* The needs, wishes, feelings and desires of the child are taken seriously by the child’s environment and the society the child lives in. There is no discrimination because of background, race or religion CRC Art. 2, 13, 14, 15, 16, 30, 37 GC 14 para. 56, 70, 73, 74, 79, 84
*3. Affective atmosphere* An affective atmosphere implies that the parents or care-providers of the child offer the child emotional protection, support and understanding. There are bonds of attachment between the parent(s) or care-giver(s) and the child. There is a relationship of mutual affection CRC Art. 19 GC 14 para. 70, 71, 72, 84	*10. Social network* The child and his family have various sources of support in their environment upon which they can depend CRC Art. 20, 37, 31 GC 14 para. 70, 73, 84
*4. Supportive*, *flexible childrearing structure* A supportive, flexible childrearing structure encompasses several aspects like: enough daily routine in the child’s life; encouragement, stimulation and instruction to the child and the requirement of realistic demands; rules, limits, instructions and insight into the arguments for these rules; control of the child’s behaviour; enough space for the child’s own wishes and thoughts, enough freedom to experiment and to negotiate on what is important to the child; no more responsibilities than the child is capable of handling CRC Art. 13, 14 GC 14 para. 70, 71, 84	*11. Education* The child receives a suitable education and has the opportunity to develop his personality and talents (e.g. sport or music) CRC Art. 17, 28, 29, 31 GC 14 para. 70, 73, 84
*5. Adequate example by parents* The parents or care-providers offer the child the opportunity to incorporate their behaviour, values and cultural norms that are important, now and in the future CRC Art. 10 GC 14 para. 70, 71, 84	*12. Contact with peers* The child has opportunities to have contacts with other children in various situations suitable to his perception of the world and developmental age CRC Art. 31 GC 14 para. 70, 73, 84
*6. Interest in the child* The parents or care-providers show interest in the activities and interests of the child and in his perception of the world CRC Art. 31 GC 14 para. 70, 71, 84	*13. Adequate examples in society* The child is in contact with children and adults who are examples for current and future behaviour and who mediate the adaptation of important societal values and norms CRC Art. 2, 8, 13, 14, 15 GC 14 para. 70, 73, 84
*Future and past*	
*7. Continuity in upbringing conditions*, *future perspective* The parents or care-providers care for the child and bring the child up in a way that attachment bonds develop. Basic trust is to be continued by the availability of the parents or care-providers to the child. The child experiences a future perspective CRC Art. 5, 6, 9, 10, 18 GC 14 para. 65, 66, 67, 70, 72, 74, 84	*14. Stability in life circumstances*, *future perspective* The environment in which the child is brought up does not change suddenly and unexpectedly. There is continuity in life circumstances. Significant changes are prepared for and made comprehendible for the child. Persons with whom the child can identify and sources of support are constantly available to the child, as well as the possibility of developing relationships by means of a common language. Society offers the child opportunities and a future perspective CRC Art. 6, 9, 10, 20 GC 14 para. 65, 70, 74, 84

Until now, research with the BIC-Model has been focused on asylum-seeking children staying in the Netherlands for several years (Zijlstra 2012). These children developed social contacts in the Netherlands, learned the Dutch language, went to Dutch schools and joined Dutch sport clubs. The disturbance of this safe and new environment would put most children at risk for damage to their development, while they had already become increasingly vulnerable while waiting for the asylum procedure to conclude. Frequent removals, related discontinuity in school careers and the emotional problems of distressed parents were identified as risk factors that contribute to the increased vulnerability of the child (Kalverboer et al. 2009).

Unlike the children residing for a longer period, the new arrivals do not yet have links with their new social environment. Therefore, they do not risk having new ties cut when they are deported. Besides that, the recently arrived children do not suffer through long periods of uncertainty, living in reception centres for years, all the while waiting for a welcome or a goodbye. However, new arrivals and longer residing children share a background in fleeing war-torn countries, exposure to violence, separations of their friends, school, family members, possessions, homes and the consequences these life events may have had on their mental health, development and well-being.

Supposing, in the case of recently arrived refugee children, that the situation shortly before the child left the country of origin will be approximately the same as the expected situation if the child would be returned soon after arrival, the analysis of these conditions for development in the home country gives decision-makers information on whether the child needs protection in the host country or which conditions need attention if a return to the home country would be the decision best serving the interests of the child.

In the next section, a systematic review of the existing knowledge in social and behavioural sciences regarding the situation of recently arrived refugee children will be presented. With this review, we aim to provide relevant elements for the assessment of the best interests of the recently arrived refugee child in a migration procedure.

## Methods

### Search Strategy

To determine relevant aspects of the best interests of the child assessment on arrival, we need to know which individual and family characteristics and which needs can be found to be of importance in the rearing environment of these children. The search strategy is based on the elements of the best interests of the child assessment, recommended by the United Nations Committee on the Rights of the Child in General Comment no. 14. The family and socio-environmental aspects of the assessment are also indicated by the conditions for development in the Best Interests of the Child-Model (Kalverboer and Zijlstra 2006; Zijlstra 2012; see introduction).

In Table 2, each aspect of the child’s best interests assessment is linked to the related search items. Whenever a search term fits more than one aspect, it is mentioned the first time only. We explored the Web of Science, PsycINFO, SOCindex, ERIC and Medline databases. Additionally, reference lists were checked. Articles published in academic journals published between 1965 and 2015 were selected.

**Table 2:** Search strategy related to General Comment No. 14 and the Best Interests of the Child (BIC)-Model

*Best interests of the child*-*aspects*	Search terms	General Comment No. 14	BIC condition
The child’s views	Views OR opinions OR ideas OR	Para. 53–54	
The child’s identity	Identity OR personality OR “evolving capacities” OR values OR traditions OR	Para. 55–57	9
Preservation of family environment and maintaining relations	Continuity OR stability OR stable OR family OR familial OR “social network” OR peer* OR relation* OR separate* OR	Para. 58–70	2, 7, 14
Care, protection and safety of the child Quality of family environment Quality of social environment	Care OR caring OR protect* OR safe* OR secure OR adequate OR integrity OR violen*OR risk* OR abuse OR wellbeing OR emotional OR physical OR affection OR degrading OR bullying OR harm OR pressure OR harassment OR exploitation OR injury OR “degrading treatment” OR conflict* OR upbringing OR “child rearing” OR parenting OR caring OR supervision OR guidance OR atmosphere OR affective OR interest OR example* OR respect OR support OR future OR perspective OR consequences OR “life circumstances” OR “living circumstances” OR	Para. 71–74	1–14
Vulnerability	Vulnerab* OR disabilit* OR disable* OR minorit* OR victim* OR resilien* OR	Para. 75–76	
Right to health	Health OR treatment OR development* OR psycho* OR psychiatric OR behavior OR	Para. 77–78, 84	1, 2, 7, 8, 14
Right to education	Education* OR school OR teach* OR learning OR capacit*	Para. 79, 84	7, 11, 14
*Age* Children	AND Child* OR young* OR adolescen*OR kid* OR minor* OR infant*		
*Background* Refugee	AND Asylum* OR refugee* OR fled OR flee OR resettle* OR “forced migrat*”		
*Timing* On arrival	AND “Recently arrived” OR “recently-arrived” OR “new arrival*” OR “on arrival”		

### Inclusion and Exclusion Criteria

Studies presenting *empirical research in social and behavioural sciences* were included, whereas review articles and studies purely about physical health have been excluded. The STROBE Statement checklist has been used as a guideline to assess the quality of the observational researches (Von Elm et al. 2007). The quality of non-observational researches was assessed by answering eighteen appraisal questions which are based on four guiding principles: (1) the research should contribute to the wider knowledge on the topic, (2) the design should be defensible, (3) the research should be rigorous by providing transparency on data collection, analysis and interpretation and (4) the research should be credible by offering well-founded arguments about the significance of the results (Petticrew and Roberts 2006, p. 152; Spencer et al. 2003).

We included studies concerning *refugee* children. The term refugee children pertains to children who were forced to leave their country of origin as a consequence of war or other harmful experiences. We excluded studies when the sample concerned migrant children without a refugee background. The included studies concern both children who have travelled to the host country alone, *unaccompanied* by their parents or other care takers, and children who fled together with (one of) their parents, referred to as *accompanied* children.

The review includes studies on *new arrivals*. Excluded were studies concerning refugee children who stay in the host country for a period longer than 1 year, or children with a residence period that was unclear.

Following the CRC, a *child* is defined as an individual under the age of 18 (Article 1, CRC). We gathered information of and insight into the situation of refugee children who came to the host country as a minor. We excluded studies concerning mixed children–adult groups whenever the results concerning the children were not presented separately. Finally, we excluded same sample studies except when other measurements were used.

**Fig. 1:**
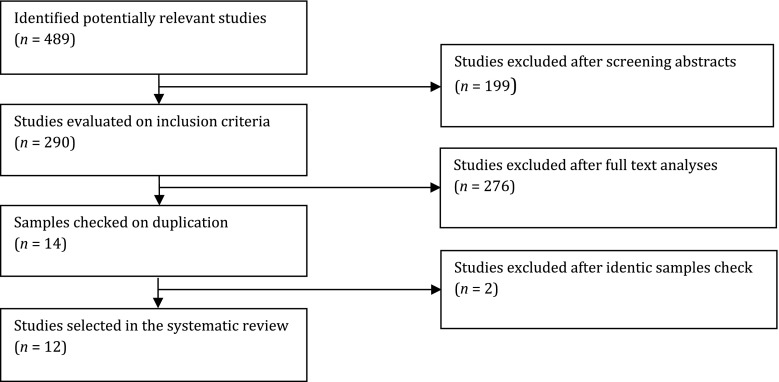
Flow diagram of study selection process

Figure 1 shows the study selection process. The database search resulted in 858 potentially relevant articles, of which 371 were duplicates. The remaining 489 abstracts were screened according to the inclusion criteria. Out of these 489 abstracts, the full text of 290 articles was reviewed. The exclusion decisions in both the abstract and the full-text reviewing phases were categorized as follows: purely physical health research (*n* = 211); no epidemiological data, reviews and comments (*n* = 110); mixed children–adults samples (*n* = 54); longer than 1-year residency (*n* = 71); and not a refugee or mixed other migrant–refugee backgrounds (*n* = 29). From the remaining 14 studies, 2 reported on the same sample. Our final selection consists of 12 studies.

## Results

### Description of the Studies

Table 3 presents the main characteristics of the included studies (*n* = 12). The summary of the outcomes is divided into descriptive and confirmatory outcomes. In the last column, significant and non-significant risk factors are separated from outcomes with a practical relevance when a striking, but non-significant influence of a risk factor was founded or the risk factors were not statistically tested.

**Table 3:** Overview of selected studies (*n* = 12)

Citation	Year	Study site	Country of origin	Number of participants	Male/ female	Age years (M)	Months since arrival	Measurements	Summary descriptive outcomes	Summary confirmatory outcomes
*Unaccompanied children*										
Jakobsen et al.^a^	2014	Norway	Afghanistan (122); Somalia (35); Iran (3)	160	160 (100 %)/0 (0 %)	14–20 (16.23)	4	HSCL-25 HTQ SLE Diagnostic interviews (CIDI)	*Experiences* 96.3 %: at least one stressful life event; mean: 6.2 (range: 0–12) 81.7 %: life-threatening events; 77.9 %: physical abuse; 77.9 %: loss of close relative; 63.3 %: drastic changes in family during the last year; 57.2 %: witnessing violence against others; 54.7 %: separated from family against will *Mental health* 41.9 %: psychiatric disorder; 30.6: PTSD; 9.4 %: depression	
Vervliet et al.^a^	2014a, b, c	Belgium (103); Norway (204)	Afghanistan (202); Somalia (47); Guinea (20); various (38)	307	291 (95 %)/16 (5 %)	15–18 (16.13)	2–5	HSCL-37A SLE RATS HTQ	*Experiences* Mean stressful life events: 6.4 (range: 0–12). 78.9 %: death of loved one; 72.5 %: physical maltreatment’; 81.8 %: experience ‘I’m in danger’; 64.0 %: drastic family changes *Mental health* 38.3 %: anxiety; 44.1 %: depression; 52,7 %: PTSD	*Significant risk factors* The more traumatic experiences the children reported, the more symptoms they had of anxiety, depression, and PTSD *Non*-*significant* Age Parents still alive
Jensen et al.	2013	Norway	Afghanistan (43); Eritrea (14); Somalia (14); Sri Lanka (11); various (11)	93	75 (81 %)/18 (19 %)	10–16 (13.8)	6	HSCL-37 SLE CPSS	*Experiences* Mean stressful life events: 5.5 (range: 0–12); 67.7 %: death of a close person; 63.4 %: witnessing violence; 62.4 %: witnessing war *Mental health* 30 %: anxiety; 20 %: depression; 54 % PTSD	*Significant risk factors* The number of stressful life events correlated with PTSD and internalizing symptoms Girls scored higher on the avoidance subscale (CPSS) *Non*-*significant* Age
Sourander	1998	Finland	Somalia (37); various (9)	46	34 (74 %)/12 (26 %)	6–17 (14.1)	5	CBCL + interviews + Clinical + legal information	*Experiences* 17 %: father disappeared; 22 %: father died; 22 %: mother disappeared; 9 % mother died; 83 %: persecution: 15 %: personal violence, 28 %: eye witnessed violence towards family members *Mental health* 48 %: clinical or borderline (related to mood, anxiety, PTSD)	*Significant risk factors* Younger children (6–14) had more severe externalizing, social and attention problems than older children (15–17) *Non*-*significant* Duration of the flight Experience of violence Gender *Practically relevant* Children coming from two-parents families were doing better that the other children
*Accompanied children*										
Sampsom and Gifford	2010	Australia	Sudan (62); Iraq (18); Ethiopia (15); various (25)	120	65 (54 %)/55 (46 %)	11–19	<12	Neighbour-hood maps, photo-novella’s + narrative data	*Well*-*being* The most important places: are associated with being able to pursue potentials largely absent in the places of their past	
Rothe et al.	2002	USA	Cuba	87	50 (57 %)/37 (43 %)	6–17 (14.9)	4–6	PTSDRI CBCL-TRF	*Experiences* 21 %: separation of father; 13 % separation of mother; 24 %: separation of sibling; 69 %: separation of grandparent(s) *Mental health* 57 %: PTSD; 67 %: avoidance; 60 % re-experiences; 52 %: somatic symptoms; 51 %: hyper-arousal	*Significant risk factors* Significant relationship between number of stressors and severity of self-reported PTSD symptoms Modest relationship between feelings that child would die at sea and witnessing violence in refugee camps with withdrawn behaviour Moderate relationship between (older) age and witnessing violence with PTSD *Non*-*significant* Gender
Abdallah and Elklit	2001	Denmark	Kosovo	1224	52 %/48 % (8 % missing data)	0–18 (8.2)	<1 (1 week)	TSF	*Experiences* 54 %: separated from one of more grandparent(s); 35 %: separated from father; 7 %: from mother; 30 %: loss close relative; 40 %: witnessing violence *Mental health* 20 %: emotional symptoms; 24 %: psychosomatic disturbances	*Significant risk factors* Increasing age was related to increasing occurrence of PTSD Duration of the flight was associated with depression, aggression and nervousness and psychosomatic problems The number of separations, number of losses and experience of torture, were associated with higher prevalence of anxiety, PTSD, depression, regressive traits and behavioural problems Extreme poverty and hunger were associated with an increasing frequency of all symptoms *Non*-*significant* Gender
Goldin et al.	2001	Sweden	Bosnia	90	46 (51 %)/44 (49 %)	0–20	6–10	Semi- structured interviews with the family + individual interviews children	*Experiences* 69 %: life was ‘good’ prior to the war; 83 %: no safe place during war; 44 % severe war experiences; 29 % direct exposure to violence; 60 %: separation from a parent *Mental health* 30 %: multiple trauma stress	*Significant risk factors* Ethnic background significantly affected the trauma stress exposure during the war. Lower social class was related to a higher intensity of child war exposure. Older children were more affected than preschool children
Geltman	2000	USA	Bosnia	31	19 (61 %)/12 (39 %)	2–17 (10.7)	<3	Bosnia War Trauma Question-naire	*Experiences* 68 %: separation from a parent; 81 %: direct exposure to armed combat; 71 %: death friend/relative; 52 %: economic deprivation *Mental health* 77 %: behavioural symptoms; 72 %: repetitive talking about violence, 52 %: nightmares; 40 % acting out; 40 % avoidance of exposure to memories	*Significant risk factors* Experiencing the death of a close relative or friend and witnessing violence to strangers were associated with re-experiencing symptoms Experiencing or witnessing interpersonal violence directed to a close relative or friend was associated with symptoms of numbing
Montgomery	1998	Denmark	Middle East: Iran (32); Iraq (168) Lebanon (22) Syria (13); stateless Palestinians (75); Turkey (1)	311	160 (51 %)/151 (49 %)	3–15 (7.5)	<1 (m 7 days)	Structured interview with parent-(s)	*Experiences* 92 %: lived in a refugee camp outside the home country; 89 %: lived under conditions of war; 89 %: been on the run with parents; 20 %: lost one parent; 60 %: separated from one parent *Mental health* 67 %: clinically anxious	*Significant risk factors* Significant predicting factors for anxiety were: lived in a refugee camp outside the home country; part of a torture surviving family; lack of opportunities for play with other children; beaten/kicked by an official; loss of father; parent hit or punished the child more than prior to arrival *Significant protective factor* Being accompanied by both parents was a modifying factor for anxiety *Non*-*significant* Age (except for separation anxiety young children after loss of father) Gender
Almqvist and Brandell-Forsberg	1997	Sweden	Iran	50	36 (72 %)/14 (28 %)	3–8 (5.10)	12	Parental interviews + children’s assessment: observa-tions, structured tasks and questions Lowenfeld World Technique	*Experiences* 84 %: exposure to violence; 32 %: eye witnessing acts of organized violence *Mental health* 68 %: behavioural symptoms (81 % of 42 who were exposed to violence); 48 %: over-dependency and anxiety (57 % of 42); 44 %: re-experiencing (52 % of 42); 18 %: PTSD (21 % of 42); 26 %: post-traumatic stress symptoms (31 % of 42)	*Practically relevant* The intensity of traumatic exposure was strongly related to the prevalence of PTSD
Ekblad	1993	Sweden	Former Yugoslavia	66	33 (50 %)/33 (50 %)	5–15	5 (m)	Structured interviews with children + parents	*Experiences* 22 %: separation of one parent; 90 % separation of a relative: ≈ 100 % experienced violence; 37 %: personal exposure to violence *Mental health* 58 %: home sick; 45 %: depression; 41 % somatic symptoms; 39 % nightmares; 28 %: fear	*Practically relevant* Experience of direct violence; apathetic or unstable mother; higher education level father; lack of proper information before flight seemed to be associated with poorer mental health

*CBCL-TRF* Child Behavioural Checklist—teacher report form, *CIDI* Composite International Diagnostic Instrument, developed by the WHO, *CPSS* Child Posttraumatic Stress Disorder Symptom Scale, *HSCL-37A* Hopkins Symptoms Checklist-37 for adolescents, *HTQ* Harvard Trauma Questionnaire, *PTSDRI* Post-Traumatic Stress Disorder Reactive Index, *RATS* Reactions of Adolescents to Traumatic Stress, *SLE* Stressful Life Events, *TSF* Trauma and Symptom Form (Danish Red Cross)

^a^The samples from Norway in the studies of Jacobsen et al. (2014) and Vervliet et al. (2014a, b, c) partly overlap. Jacobsen et al. (2014) added a diagnostic interview to the measurements, which provided more results on the sample. For this reason, both studies were included

All together, the studies concerned 2585 children. Out of these 2585 children, 1979 were accompanied by their parents on arrival (*n* = 8) and 606 children were unaccompanied (*n* = 4). In the studies of unaccompanied children, the most prevalent countries of origin were Afghanistan (367 children) and Somalia (133 children). The remaining 106 children came from a range of countries.

From the eight included studies of accompanied children, the majority (*n* = 6) concerned children from one country or region: former Yugoslavia (*n* = 4), Iran (*n* = 1) and Cuba (*n* = 1). Except for one, all of these studies presented descriptions and calculations of adverse experiences that the refugee children had been exposed to and connected these to mental health problems (*n* = 11). One study focused on places that contribute to the recovery and well-being of recently arrived refugee children.

### Stressful Life Experiences of Refugee Children Before Arrival in the Host Country

#### Unaccompanied Children

Three studies used the Stress Life Events scale (SLE) to identify the *number of stressful life experiences* of the children before arrival in the host country. Of the 12 events mentioned in the SLE, the children reported an average of 5.5–6.4 stressful events (Jakobsen et al. 2014; Vervliet et al. 2014b). The average number of stressful life events in a Dutch (non-clinical) reference group was three (Bean et al. 2004).

Children who arrive in the host country on their own have experienced the separation from their parents by definition. About three quarters of the unaccompanied refugee children experienced both the *disappearance and loss* of close relatives. Approximately half of these children experienced a drastic change in the family situation during the last year (Jakobsen et al. 2014; Jensen et al. 2013; Vervliet et al. 2014b).

The vast majority of the unaccompanied children have previously been exposed to *violence*, life-threatening events (Jakobsen et al. 2014; Jensen et al. 2013; Vervliet et al. 2014b) or persecution (Sourander 1998). Half of these children have been exposed to war and witnessed violence or life threats against others (Jakobsen et al. 2014; Jensen et al. 2013; Vervliet et al. 2014b). Sourander (1998) reported 28 % of the children to have witnessed violence (e.g. rape, torture and physical violence) done to their parents.

#### Accompanied Children

Four of the eight studies included in our review concerned accompanied children in former Yugoslavia in the nineties of the last century and provided an account of their experiences during the war (Abdalla and Elklit 2001; Ekblad 1993; Geltman et al. 2000; Goldin et al. 2001). Approximately 80 % of the Bosnian children have been exposed to war violence, such as grenade explosions, random bombings or gunfire (Ekblad 1993; Geltman et al. 2000). Separation from and loss of close family members are common among these children (Abdalla and Elklit 2001; Ekblad 1993; Geltman et al. 2000). Torture, injury or the killing of a close relative has been experienced by 35 (Geltman et al. 2000)–40 % (Abdalla and Elklit 2001) of the children. The number of traumatic events could not be assessed in these studies of *war experiences*, since the violence was ongoing for extended periods of time (Geltman et al. 2000).Goldin et al. (2001) clustered the war-related stories of 90 refugee children and their families from Bosnia concerning trauma and stress factors prior, during and after war. Prior to the war, life was “good” for the vast majority (62/90) of the children, characterized by strong family ties, friends and school, which made life meaningful and predictable. The most severely affected group consisted of 26 children who have had violent war experiences and endured persecution directed to the child’s home or family. Separation from a parent occurred most often in this group (22/26) (Goldin et al. 2001). Hunger and extreme poverty were prevalent among the Kosovarian refugee children (Abdalla and Elklit 2001). The experiences of children coming from war zones in the Middle East bear a resemblance to those of the Bosnian and Kosovarian children. In Montgomery’s research (1998), 89 % of the 311 refugee children from the Middle East (Iran, Iraq, Lebanon, Syria, Palestinians) had lived in war conditions; 90.8 % had to take shelter for bombing, and 86.4 % had been on the run with their parents; 68.2 % witnessed violent events such as bombings (82.6 %), street shootings (68.8 %) or had their house searched (60.5 %). One out of five (19.9 %) of these children has experienced the death or disappearance of a parent, and 59.5 % has been separated from a parent for more than 1 month.

Children from Iran were exposed to both individual *persecution* and general war violence. Iranian parents reported that 84 % of their children had been exposed to violence. They were eyewitnesses of acts of organized violence, such as a violent raid of their home or assault on a parent (Almqvist and Brandell-Forsberg 1997).

In a study about Cuban refugee children, the children seemed to be mostly affected by the *dangerous flight* itself. These children fled in the mid-nineties mostly by boat (50 %) or on a home-made raft (38 %). About 34,400 Cuban people were intercepted by the US Coast Guard and brought to detention camps. Both the ocean crossing and the stay in the detention camps were a huge stress factor for the children. One-third (30 %) of these children thought they would die during the crossing and 80 % witnessed acts of violence in the camps (Rothe et al. 2002).

### Mental Health Problems of Recently Arrived Refugee Children

#### Unaccompanied Children

The four selected studies on recently arrived unaccompanied refugee children focused on mental health problems, and all four found that approximately half of the children faced such problems. Sourander (1998) found that nearly half of the unaccompanied minors in his research had behavioural problems in the clinical or borderline range. The most common symptoms were related to *PTSD*, *depression and anxiety*. In the other three studies, between one-third and half of the children were diagnosed with *PTSD.* Furthermore, anxiety and depressions were the most prevalent symptoms (Jakobsen et al. 2014; Jensen et al. 2013; Vervliet et al. 2014b).

#### Accompanied Children

All studies focusing on the mental health of recently arrived accompanied children (*n* = 7) reported high levels of *traumatic stress or emotional symptoms* in general terms (Abdalla and Elklit 2001; Almqvist and Brandell-Forsberg 1997; Goldin et al. 2001) or *PTSD* (Almqvist and Brandell-Forsberg 1997; Rothe et al. 2002). In one research, three quarters of the children showed repetitive talking about violence (Geltman et al. 2000). Nightmares were reported in 39–52 % (Ekblad 1993; Geltman et al. 2000). Avoidance of exposure to memories was seen in 40–67 % of the children (Geltman et al. 2000; Rothe et al. 2002) and re-experiencing of traumas in nearly half of the children (Almqvist and Brandell-Forsberg 1997).

Of the 311 children in Montgomery’s (1998) research, two-thirds were identified as being clinically anxious. The most frequently reported symptoms of anxiety were: “fear of sleeping without light”, “fear of being alone” and “clinging to parents”. In the research of Rothe et al. (2002), separation *anxiety* and clinging to parents were classified as the most severe symptoms observed by the researchers. In another research, half of the children were diagnosed to be suffering from anxiety (Almqvist and Brandell-Forsberg 1997).

One study mentioned that nearly half of the children were diagnosed with *depression* (Ekblad 1993).

In two studies, mental health problems were described as *behavioural symptoms*; the prevalence ranged from 68 to 77 % (Almqvist and Brandell-Forsberg 1997; Geltman et al. 2000).

The prevalence of *psychosomatic symptoms* ranged from 24 to 52 % (Abdalla and Elklit 2001; Ekblad 1993; Rothe et al. 2002).

One study reported 58 % prevalence of *homesickness* (Ekblad 1993).

### Risk and Protective Factors

#### Unaccompanied Children

Children who were exposed to a higher *number of adverse life events* are at a higher risk of having PTSD symptoms and internalizing problems such as depressions and anxiety (Jensen et al. 2013; Vervliet et al. 2014b).

In the research of Sourander (1998), the younger group (6–14) had significantly more severe behavioural problems than the older group (15–17). Sourander suggests that this may be explained by the fact that older children possess more internal resources to cope with such stressful experiences. However, the other included studies did not find *age* to have a significant effect on mental health problems (Jensen et al. 2013; Vervliet et al. 2014b).

A child’s *gender* was not a significant factor for the mental health problems these children were facing or for the number of stressful life events these children reported (Jensen et al. 2013; Vervliet et al. 2014b).

#### Accompanied Children

The *numberof stressful life events* (Rothe et al. 2002) and the *duration of separation with parents* experienced by these children are associated with the occurrence of PTSD (Abdalla and Elklit 2001). *Exposure to violence* (Abdalla and Elklit 2001; Ekblad 1993; Rothe et al. 2002), and more specifically, the *intensity* (Almqvist and Brandell-Forsberg 1997) and *duration* (Montgomery 1998) of the exposure to violence, the *losses of close relatives* (Montgomery 1998) and *extreme poverty* (Abdalla and Elklit 2001) are all associated with increased occurrence of depression, aggression, nervousness, behavioural problems and PTSD.

The *durationof the flight* is linked to the number of losses and separations that these children experience, and these events are, as described above, risk factors for mental health problems (Abdalla and Elklit 2001). The *feeling of being in danger* during the flight is associated with withdrawal behaviour (Rothe et al. 2002). One study also described the *lack of information* given to the children by their parents concerning their flight as a possible risk factor for mental health problems (Ekblad 1993). Further, *living in a refugee camp* has also been identified as a risk factor (Montgomery 1998).

Two studies found that older children have an increased risk of suffering from PTSD (Abdalla and Elklit 2001; Rothe et al. 2002). Two studies mentioned that teenagers faced more severe traumatic experiences during the war due to their longer life but also because of the fact that they were more out going than younger children (Abdalla and Elklit 2001; Goldin et al. 2001). However, *age* was not considered to be a significant variable in other studies (Geltman et al. 2000; Montgomery 1998).

During the war in Bosnia, children with a Bosniak (Bosnian Muslim) *ethnic background* more severely suffered traumatic experiences, compared to children with a Bosnian Croat or Serb ethnicity (Goldin et al. 2001).

*The role of the mother* seemed to be both a risk and protective factor in Ekblad’s study (1993). She states that children with an apathetic or unstable mother are at an increased risk, whereas children with a more optimistic mother are at a lower risk of developing mental health problems. Goldin et al. (2001) described how children from a lower *social class* were significantly more often exposed to severe war incidents than children from a higher class, which had better opportunities to reach a safe place. Ekblad (1993), on the other hand, reported higher education of a father to be risk factor, which she thought could be explained by the probability of a higher level of frustration. The *current behaviour of parents* towards children was a risk factor for anxiety when one or both parents hit and/or punished the child more often in the host country than in the country of origin. This behaviour was presumed to give the child feelings of rejection (Montgomery 1998). Arriving in the company of both parents was a modifying factor for anxiety (Montgomery 1998).

Sampson and Gifford (2010) explored the *significance of certain places for the well*-*being* of young refugees. The most important place for the refugees was considered to be their own home, their school, the local parks and libraries. In their study, Sampson and Gifford analysed the specific contribution of these places to the well-being of young refugees. Places of opportunity promoted the meaning and purpose of life. Places of restoration reduced fear and anxiety and promoted dignity and value. Places of sociality helped the youth to restore relationships and promoted attachment and connection to others. The last category, places of safety, helped the young refugees to get a sense of security.

## Discussion

### Elements for the Best Interests of the Child Assessment

#### Factors of Vulnerability

The determination of vulnerability factors is an inherent part of the best interests of the child assessment (GC 14, para. 75–76): before a decision in a migration decision can be taken, the vulnerability of the refugee child should be assessed. Our systematic research of the situation of newly arrived refugee children has shown that it is important to know which and how many stressful life events a child has experienced before arrival in the host country, as well as the duration and severity of these events. Studying these events is not only important to determine the reason why a child asks for protection, but also because these events constitute risk factors for the mental health of the child. Relevant experiences that should be taken into account in this process are exposure to violence, separation and loss of close relatives, feelings of being in danger prior to and during the flight, family situational changes, physical maltreatment, extreme poverty and the circumstances of life in a refugee camp outside the home country.

The fact that minor refugees have been exposed to a range of traumatic experiences on arrival in the host country calls for special consideration in the assessment procedure. The accumulation of risk factors is associated with an increased likelihood of children acquiring developmental problems (Caprara and Rutter 1995; Rutter 1979).

The most common mental health problems children face upon arrival are PTSD, depression and several anxiety disorders. It is essential that these problems are addressed at an early stage, since we know that young refugees still struggle with mental health problems even after spending a significant time in the safe environment of the host country (Almqvist and Broberg 1999; Bean et al. 2007b; Bronstein et al. 2012; Oppedal and Idsoe 2012; Seglem et al. 2011; Vervliet et al. 2014a). These problems may portend that the refugee child’s issues persist after arrival, or that new experiences in the host country, such as feelings of uncertainty about the outcome of the migration procedure and frequent relocations, put the children at risk again (Bean et al. 2007b; Nielsen et al. 2008). This accumulation of stress factors has a detrimental effect on the mental health of minor refugees (Bronstein and Montgomery 2011) and should be considered to be an important element of the best interests of the child assessment in the migration procedure.

#### Lack of Information of Family and Social Context

In General Comment No. 14, the UN Committee on the Rights of the Child states that, in addition to the individual characteristics of the child, the social-cultural context of the child should also be included in a best interests of the child assessment (GC 14, para. 98). In this assessment, the preservation of the family environment and the possibility of maintaining relations with kin are guiding principles (GC 14, para. 58–70), and care, protection and safety for the child should be the primary focus (GC 14, para. 71–74). The Best Interests of the Child (BIC)-Model is a pedagogically underpinned translation of how the family and social environment of the child, which, of course, can also be applied to children in the migration context (Kalverboer 2014; Kalverboer and Zijlstra 2006; Zijlstra 2012). We propose that the fourteen conditions for development (Table 1) should be assessed for each child that asks for international protection. None of the included studies provided an in-depth view on this important subject. Only Montgomery (1998) included a few items concerned with the rearing environment of the child. It can be concluded that when looking at the situation upon arrival, next to nothing is known of the rearing environment of minor refugees. This is a major concern, since it is impossible to make a decision in the best interests of the child about his or her request for protection in the host country, without an assessment of the protective capacity of the child’s environment. Therefore, further research on this subject is needed.

Although unaccompanied children arrive in the host country without their parents, their family conditions should be assessed as well. For both recently arrived unaccompanied children and accompanied children, the situation prior to the flight is crucial in the best interests of the child assessment, since that is where the child will return to in case his/her request for protection is denied. Prior to their flight, most unaccompanied children probably lived somewhere with their family members. Therefore, an assessment of their capacity to provide a safe environment and protect the development of the child is also necessary. With this, the BIC-model might prove helpful.

#### Fit with Previous Systematic Reviews

Two systematic reviews (Bronstein and Montgomery 2011; Fazel et al. 2012) of the mental health of longer residing refugee children confirm the previously mentioned risk factors for the mental health of recently arrived children. These reviews found three additional relevant factors that are related to the pre- and on-arrival situation of the children: pre-existing vulnerability, being unaccompanied and poor parental support and cohesion.

In a longitudinal research, pre-existing vulnerability (delayed development, long-term physical illness or psychological health problems) appeared to be a risk factor for the mental health of refugee children (Almqvist and Broberg 1999). This aspect should be included in the description of the vulnerability in best interests of the child assessment.

Neither the stressful life events, nor the type and prevalence of mental health problems differed unambiguously between accompanied and unaccompanied minors in our review. This result contrasts the fact that being an unaccompanied minor has been identified as risk factor for mental health problems in various studies and reviews (Bean 2006; Bean et al. 2007a, b; Bronstein and Montgomery 2011; Derluyn et al. 2008; Fazel et al. 2012; Hodes et al. 2008). First of all, the instruments and definitions that were used in the included studies concerning unaccompanied and accompanied children were different; for that reason, a meta-analysis of the data was impossible. Moreover, the absence of a clear difference between unaccompanied and accompanied minors in the studies selected may be explained by the short period of residence in the studies’ samples. Forced migration is associated with loss and separation for all refugee children, but missing one’s parents may impact the mental health of unaccompanied minors in the long term more severely. Also, the UN Committee on the Rights of the Child does recognize *unaccompanied* minor refugees as vulnerable children (General Comment No. 6, para. 1) who are entitled to appropriate protection (Article 22, CRC).

In summarizing Table 4, we connect the various risk factors found in our own review and in previous systematic reviews to the elements of the best interests of the child assessment, based on General Comment No. 14 of the UN Committee on the Rights of the Child and the Best Interests of the Child-Model.

**Table 4:** Elements of the best interests of the child assessment based on General Comment No. 14 of the UN Committee on the Rights of the Child, the Best Interest of the Child-Model and risk factors (*italic*)

Individual characteristics		Family and Social environment	
Identity	Situation of vulnerability	Conditions for development in the family	Conditions for development in the society
Elements of the best interests of the refugee child assessment upon arrival			
Gender Sexual orientation Nation of origin Religion and beliefs Cultural Age	Being refugee, asylum seeker, migrant *Pre-existing vulnerability: development*, *illnesses*, *extreme poverty* *Being unaccompanied* *Number*, *severity and duration of stress full life events* *Exposure to violence* *Witnessing violence* *Dangerous and/or long flight* *Having stayed in refugee camps* (*Number of*) *separations* *Mental health: PTSD*, *anxiety*, *depression*	Care, protection, safety of the child and rearing Conditions within the family *Extreme poverty* *Poor parental support* *Dead and losses of close relatives* *Separations*	Safe environment *Exposure to violence* *Witnessing violence* *Feeling of being in danger* Social environment *Experience of discrimination* *Lack of social support* *Lack of opportunities to play* Education
Prospects in the future			
Possibility to preserve identity	Possibility to address special needs, including (mental) health care	Preservation of the family environment *Drastic changes in family* *Dead and losses of close relatives* *Separations* *Poor parental support*	Stability and future perspectives in society on safety, protection, possibility to address educational needs, preservation of social ties

The child’s views on all elements and on his/her need for protection

### Strengths and Limitations

The strength of this study is that by using a search strategy on all relevant elements of the best interests of the child assessment for recently arrived refugee children, our study provides an overview of the current knowledge in behavioural and social sciences of the situation of the refugee child; something that, to our knowledge, has not been done before. At the same time, given that the number of studies on this specific situation is limited, the results have to be interpreted with caution.

We have seen studies that failed to provide a clear statement concerning the period of time that the refugee children in the study sample resided in the host country. This may have led to missing articles in the review. We have chosen to be strict about the elapsed time since arrival (<1 year) in order to get a clear picture of the currently existing knowledge about the well-being and development of refugee children at the moment of their arrival in the host country.

Most studies about longer residing refugee children additionally include information on the pre-migration period. However, this retrospective information is not included in this research because of the time exclusion criterion. Yet, risk factors that occur upon arrival and may have a long-term impact on the mental health of the refugee child should also be taken into account. We addressed this limitation by comparing our results to those of the systematic reviews of the mental health of longer residing refugee children.

### Implications for Assessment of the Best Interests of the Child

This systematic review sheds light on which stressful life events, mental health problems and risk factors have proven to be relevant for an assessment of the vulnerability of the child (Table 4). The exposure to stressful experiences and the high prevalence of mental health problems among these children underlines the need to involve professionals with knowledge of child development and child psychology during the best interests assessment, as the UN Committee on the Rights of the Child prescribes in General Comment No. 14 (para. 94). Decision-making in the migration procedure may be facilitated by using this expert knowledge (Steel et al. 2004).

### Implications for Interviewing Refugee Children

The views of the child are an inherent part of the assessment, in order to ensure the influence of the child on the best interests determination (GC 14, para. 53). The United Nations Committee on the Rights of the Child (2009) provided guidelines on a child’s right to be heard. The fact that the child is in a vulnerable situation because of, for instance, their migrant status “… does not reduce the weight given to the child views in determining his or her best interests” (GC 14, para. 54). None of the included studies reported on the views of the children on their residence procedure. To make a decision in the migration procedure of recently arrived refugee children, these views have to be gathered. In addition, it is important to ask the children about their personal and their family’s migration motives, in order to get a picture of the aspirations of the child and any expectations others may have of the child’s stay in the host country Vervliet et al. 2014a, b, c.

Interviewers in the decision-making procedure should be aware that the traumatic experiences may hamper the ability of refugee children to tell their story in a coherent and consistent manner (Evans Cameron 2010; Herlihy et al. 2002; Herlihy and Turner 2006; Spinhoven et al. 2006; UNHCR 2013, 2014). Apart from the effect of traumatic experiences, interviewers of refugee children may meet additional difficulties as a result of mistrust and its subsequent silence which are often seen among young refugees (Anderson 2001; Adams 2009; Björnberg 2011; Chase 2010; De Haene et al. 2010; Ghorashi 2008; Hynes 2009; Kelly 2012; Kohli 2006a; 2006b; 2011; McKelvey 1994; Miller 2004; Ní Raghallaigh 2014).

More profound knowledge on how refugee children can be supported to reveal their life stories is needed. Research in the field of mental health care, social work and asylum procedures has revealed some relevant facilitators that could be helpful, like a positive and respectful attitude of the interviewer and using non-verbal methods to support verbal narrative telling (Van Os et al. 2016).

### Implications for Protection Grounds for Refugee Children

The knowledge of recently arrived refugee children in behavioural and social sciences provides research-informed guidelines on the elements that have to be taken into account when taking a decision in a migration procedure. This knowledge may seem to be just partly relevant in the context of asylum. Decisions in asylum procedures concentrate on the issue of “well-founded fear of being persecuted” (Article 1A, 1951 Convention Relating to the Status of Refugees, UNHCR 1951). Taking the best interests of the child as a primary consideration implies looking at the asylum request through “child rights glasses”. This means that violations of child-specific rights should be assessed; that the decision-makers should be aware of the fact that children may experience harm differently than adults; and that child-specific forms of persecution have to be taken into account (UNHCR, 2009). If a child is not accepted as a refugee, there still has to be made a decision in the best interests of the child concerning the place where he or she can live. All elements described in this paper have to be taken into account when taking such a decision. Migration policy based on children’s rights may require alternative answers when children’s rights are at stake (Bhabha 2014; Drywood 2011; Evenhuis 2013; McAdam 2006).

We believe that a decision about the child’s need for international protection could be based on the child’s right to development, similarly to the way it is being applied nowadays in child protection law. If a child’s development is at risk in his or her current living situation, the State authorities have an obligation to intervene in order to safeguard the safety and development of the child (Articles 6 jo. 19, CRC). For unaccompanied refugee children, the Convention on the Rights of the Child requires looking at regular national child protection systems (Article 22, Sect. 2, CRC) in order to safeguard the “appropriate protection” these children are entitled to (Article 22, Sect. 1, CRC). For both accompanied and unaccompanied children, this obligation can be derived from the non-discrimination principle (Article 2, CRC), combined with the articles on child protection, when the development of a child is endangered (Articles 6 jo. 19, CRC). All things considered, during the assessment of the best interests of the child in a migration procedure, either resulting in a residence permit or in a return decision, the core principle should be to treat refugee children in the same way as any other children at risk.
